# Evidence for SARS-CoV-2 Infection of Animal Hosts

**DOI:** 10.3390/pathogens9070529

**Published:** 2020-06-30

**Authors:** Ahmed S. Abdel-Moneim, Elsayed M. Abdelwhab

**Affiliations:** 1 Microbiology Department, Virology Division, College of Medicine, Taif University, Al-Taif 21944, Saudi Arabia; asa@tu.edu.sa or; 2 Virology Department, Faculty of Veterinary Medicine, Beni-Suef University, Beni-Suef 62511, Egypt; 3 Institute of Molecular Virology and Cell Biology, Friedrich-Loeffler-Institut, Federal Research Institute for Animal Health, 17493 Greifswald-Insel Riems, Germany

**Keywords:** SARS-CoV-2, Coronaviridae, COVID-19, pandemic, viral zoonosis, interspecies transmission, bats, animal modeling, zoo animals, pets

## Abstract

COVID-19 is the first known pandemic caused by a coronavirus, SARS-CoV-2, which is the third virus in the family Coronaviridae to cause fatal infections in humans after SARS-CoV and MERS-CoV. Animals are involved in the COVID-19 pandemic. This review summarizes the role of animals as reservoirs, natural hosts and experimental models. SARS-CoV-2 originated from animal reservoir, most likely bats and/or pangolins. Anthroponotic transmission has been reported in cats, dogs, tigers, lions and minks. As of now, there is no a strong evidence for natural animal-to-human transmission or sustained animal-to-animal transmission of SARS-CoV-2. Experimental infections conducted by several research groups have shown that monkeys, hamsters, ferrets, cats, tree shrews, transgenic mice and fruit bats were permissive, while dogs, pigs and poultry were resistant. There is an urgent need to understand the zoonotic potential of different viruses in animals, particularly in bats, before they transmit to humans. Vaccines or antivirals against SARS-CoV-2 should be evaluated not only for humans, but also for the protection of companion animals (particularly cats) and susceptible zoo and farm animals.

## 1. Coronaviruses

### 1.1. Classification of Coronaviruses

Coronaviruses (CoVs) belong to the order Nidovirales, suborder Cornidovirineae, family Coronaviridae and subfamily Orthocoronavirinae. The latter is composed of four genera designated alpha, beta, gamma and delta CoVs (α-, β-, γ- and δ-CoV), corresponding to groups one to four (I to IV), respectively. This classification is based on sequence analysis, phylogenetic relatedness and serologic examination [1]. 

#### 1.1.1. Animal Coronaviruses

Bats and birds are major reservoirs for CoVs [2,3]. New diversified CoVs have been identified worldwide [2,4], and several γ- and δ-CoVs have been isolated from wild and domestic birds (e.g., geese, pigeons, ducks, bulbuls, thrushes and munias) [3]. Under natural conditions, each CoV has a narrow restricted host range infecting a single animal species, and interspecies transmission occurs rarely, if at all [5]. They mostly infect respiratory and/or digestive tracts, and few viruses can spread to the kidneys, liver or central nervous system. Some CoVs are endemic in domestic animals in different countries [5]. The most common members of CoVs infecting animals are infectious bronchitis virus (IBV; γ-CoV) in chickens [6]; porcine transmissible gastroenteritis coronavirus (TGEV; α-CoV) [7], porcine hemagglutinating encephalomyelitis coronavirus (HEV; β-CoV) [8] and porcine epidemic diarrhea coronavirus (PEDV; β-CoV) [9] in swine; bovine CoV (BCoV, β-CoV) in cattle [10]; canine enteric coronavirus (CECoV; α-CoV) [11] and canine respiratory coronavirus (CRCoV; β-CoV) [12] in dogs; feline coronavirus (FCoV; α-CoV) in cats [13]; and murine hepatitis virus (MHV; β-CoV) in mice [14] (Table 1).

#### 1.1.2. Human Coronaviruses (HCoVs)

There are four established coronaviruses (HCoVs) that can infect humans, namely HCoV-E299 (α-CoV), HCoV-NL63 (α-CoV), HCoV-OC43 (β-CoV) and HCoV-HKU1 (β-CoV) (Table 1). Human infections with any of these four viruses are relatively mild and constitute 10–30% of the causative agents of the common cold in humans [15,16,17]. Nevertheless, the infection with HCoVs can cause lower respiratory tract infections (LRTIs), with serious consequences in the young, the elderly and immunocompromised individuals [18]. Although HCoV-NL63 and HCoV-HKU1 have been in circulation for a long time in several countries, they were only discovered after 2003, in the post-SARS era [19]. Interestingly, HCoV-OC43 and bovine BCoV shared 95% genetic identity, indicating zoonotic transmission from cattle to humans 100 years ago. Bovine-to-human, and not human-to-bovine, transmission of HCoV-OC43 is supported by the presence of a 290-nucleotide deletion in HCoV-OC43, which was absent in BCoV, suggesting adaptive changes after jumping the species barrier to humans [20]. Moreover, human infections with three animal origin β-CoVs, namely HCoV-SARS, HCoV-MERS and the most recent CoV, SARS-CoV-2, were reported to induce severe LRTIs in humans [1]. Certain animals (see below) are also vulnerable to SARS-CoV-2 because the same Angiotensin-Converting Enzyme 2 (ACE2) receptor is used, and this receptor is quite conserved across mammals [21,22,23].

### 1.2. Coronavirus Structure and Genome Organization

The coronavirus particle is conserved across the observed diversity of these viruses [42]. The surface of the virion possesses club-shaped spike projections, giving the virus the appearance of a solar corona. The RNA genome of coronaviruses is the largest known genome of RNA viruses [43]. It is positive-sense linear single-strand with 26–32 kb. The genome is typically organized as 5′-leader-UTR–replicase-S (Spike)–E (Envelope)-M (Membrane)-N (Nucleocapsid)-3′UTR-poly (A) tail with many accessory genes [43]. The genome encodes structural, accessory and non-structural proteins. The structural proteins include spike (S), envelope (E), matrix (M), nucleocapsid (N) and some CoVs, which express hemagglutinin-esterase (HE), apparently derived from influenza C viruses [44] (see Table 2). The viral envelope is studded by the S, E, M and HE [45,46]. The S protein possesses receptor-binding domain (RBD), antigenic epitopes and cleavage site (CS). The S protein is cleaved by host proteases into S1 and S2 subunits, which are responsible for binding to the host cell receptor and fusion of viral and cellular membranes, respectively. The receptors of CoV are variable and mostly virus-specific (Table 2). The M protein is a transmembrane protein. It is the most abundant structural protein and is important for virus morphology. The E protein is expressed in smaller amounts than the other structural proteins, and it plays roles in assembly and release of the virus and has an ion-channel activity, while the N protein encapsidiates the viral RNA genome. The replicase gene is about 20kb (two-thirds of the genome) and encodes two open reading frames, ORF1a and ORF1b, which express two polyproteins, pp1a and pp1ab, respectively; the latter requires frameshifting by the polymerase [47,48]. Subsequently, pp1a and pp1b cleaved into individual non-structural proteins (nsps 1 to 16) after the expression of papain-like proteases (PLpro), encoded within nsp3 and a serine-type protease or Mpro encoded by nsp5 [49,50]. Furthermore, many of these nsps assemble into the replicase–transcriptase complex (RTC) responsible for RNA replication and transcription, including, for example, the RNA-dependent RNA polymerase (RdRp; nsp12) [51]; the RNA helicase, 5′-triphosphatase (nsp13) [52], the N7 MTase and 3′-5′ exoribonuclease (ExoN) (nsp14) involved in replication fidelity and N7-methyltransferase activity [53] and 2′-O-methyltransferase (nsp16) [54]. The accessory proteins are mostly dispensable for virus replication in cell culture; however, they might be essential for viral pathogenesis [43,55]. Some accessory proteins also play a role in blocking innate immune responses, e.g., nsp1, which is absent in γ-CoV (avian infectious bronchitis virus “IBV” and turkey coronavirus “TCoV”; see Table 1). This is likely why they are non-essential for replication [43,56]. 

### 1.3. Genetic Evolution of Coronaviruses

Coronaviruses (CoVs) evolve through recombination and point mutations. The large viral RNA genome coupled with low fidelity of RdRp (nsp12) allows the occurrence of spontaneous mutations during virus replication, although at lower rates than other RNA viruses [57,58,59], because CoVs have a proofreading mechanism which seems to cause the lower substitution rate compared to other RNA viruses [60]. The mutation rate of CoVs is variable. For example, the mutation rates of murine hepatitis virus (MHV) and IBV have been estimated to be 0.44–2.77 × 10^−2^ and 0.67–1.33 × 10^−5^ per site per year, respectively [61,62], while the evolution rate of SARS-CoV-2 was estimated to be ~9 × 10^−4^ substitution per site per year [63]. Moreover, the mutation rate of CoVs can be increased more than five times under immune pressure (e.g., vaccination) or upon interspecies transmission [4,58,64,65,66,67,68]. Importantly, CoVs are subjected to high-frequency recombination events with rates of about 20% during mixed infection of cells with closely related viruses [69]. Such “mosaic” recombination was responsible for the natural evolution of novel viruses as reported in SARS-CoV [70,71] and MERS-CoV [72] in addition to other CoVs [73,74,75]. In vitro, the generation of chimeric coronaviruses with high replication efficiency in human cells has been frequently described [76,77,78,79,80]. Such findings confirm the possibility of natural recombination in the emergence of potential pathogens to humans [78]. Therefore, recombination of the virus genome is a major pathway for the evolution of CoVs with efficient interspecies or intraspecies transmission capacity or higher virulence [81,82,83,84].

## 2. SARS-CoV-2

In December 2019, a cluster of human cases of severe pneumonia of unknown etiology was detected in Wuhan, Hubei Province, China. The infection has been traced back to seafood and a wet live animal wholesale market in the city [85]. On the 7th of January, a novel CoV was identified as the causative agent. Different tentative names, including novel Coronavirus 2019 (nCoV-2019) and 2019-nCoV, have been proposed for the new virus [85]. On 11 February, the WHO named the disease as coronavirus disease-2019 (COVID-19). The international committee for taxonomy of viruses (ICTV) published the official nomenclature of the virus as SARS-CoV-2 [86]. On 29 February, the WHO declared that the disease is called COVID-19 and the virus that causes it is SARS-CoV-2 [87]. On 11 March, the virus was declared as a pandemic, marking the first known pandemic caused by a coronavirus [88]. In this review, we summarize data on SARS-CoV-2 in animals, available on 1 June 2020, in PubMed, Google Scholar, preprint servers and websites of animal and human health organizations (e.g., OIE, CDC and USDA).

### 2.1. Animal Hosts

#### 2.1.1. Origin of SARS-CoV-2 and Wild-Animal Reservoir

The identification of reservoirs of zoonotic pathogens often plays a crucial role in effective disease control. Zoonotic pathogens, which can infect a wide range of hosts (e.g., influenza), have been demonstrated as a serious risk factor for emergence and re-emergence in humans [89,90]. The majority of significant viral diseases of humans have been transmitted from domestic and/or wild-animal reservoirs (reviewed in References [91,92,93,94,95,96,97]). Although we may never know with certainty the precise route of transmission of SARS-COV-2, it is widely accepted that SARS-CoV-2 has an animal origin. However, it remains to precisely identify the animal reservoir(s). Bats were the reservoir for SARS-CoV (2003–2004) [98] and diverse SARS-related CoVs (SARSr-CoVs) [79,99]. Therefore, it is most likely that bats are the current potential reservoir for SARS-CoV-2 [4], which was genetically close to a horseshoe bat SARSr-CoV (designated RaTG13), with 96% genetic similarity [100]. This virus was isolated from *Rhinolophus affinis*, between 2015 and 2017, from Yunan Province, which is located far away from Wuhan (about 2000 km) [100,101]. However, the RBD and CS in the S protein are distinct between SARS-CoV-2 and RaTG13. The latter has a monobasic CS and several mutations in the RBD, compared to the SARS-CoV-2. Extensive sequence analysis estimated that RaTG13 and SARS-CoV-2 diverged 40 to 70 years ago, most likely in horseshoe bats [102]. Recently, a novel SARSr-CoVs (designated RmYN02) with an insertion of polybasic amino acids in the CS was detected from the Yunnan Province, between May and October 2019 [103]. With testing more samples from bats in China, there is a possibility to identify more related strains to SARS-CoV-2. Moreover, involvement of other intermediate hosts, probably pangolins, as a plausible conduit in the transmission of SARS-CoV-2 to humans cannot be excluded [102]. Recent studies found that Malayan pangolins (*Manis javanica*) are frequently infected with CoVs. Diverse CoVs identified in the lungs, intestine and/or blood of pangolins sampled in 2017–2018. Sequence analysis indicated that pangolin-CoVs belonged to two different lineages, and one lineage shared 97.4% amino acid identity to RBD with SARS-CoV-2. Therefore, pangolins are considered to be a potential intermediate host for SARS-CoV-2 [102,104,105,106,107]. Up to the end of May 2020, little to no evidence of recombination was observed [108]; however, it is conceivable that SARS-CoV-2 evolved after multiple “mosaic” recombination events of bat and/or pangolin SARSr-CoV. The currently available data do not rule out a non-pangolin or bat intermediate host. 

#### 2.1.2. Natural Infection in Animals

##### Dogs

In a surveillance of 27 dogs in Hong Kong, two dogs tested positive [109,110,111]. The first dog was identified on February 27, 2020 [109,110,111]. SARS-CoV-2 RNA was detected in swabs in the nasal and oral cavities of a quarantined 17-year-old Pomeranian dog. The owner tested positive for the virus, suggesting a human-to-dog transmission. The virus titer was very low in the dog samples, and no clinical signs were observed. Genetic analysis revealed that the dog and human viruses were closely related, indicating possible human-to-dog transmission. A few days later, neutralizing antibodies were detected in the blood samples. The dog died after three days from the quarantine, probably due to unrelated health issues, rather than SARS-CoV-2 infection [109,110,111]. The second dog was identified on March 18, 2020 [109,110,111]. A 2.5-year-old asymptomatic German shepherd dog tested positive for SARS-CoV-2 RNA, and neutralizing antibodies developed a few weeks later. The dog probably acquired the infection from the owner, who was also infected with the virus [109,110,111]. In France, neither RNA nor antibodies were detected in dogs living in the same room with veterinary students infected with SARS-CoV-2 [112]. Likewise, viral RNA was not detected in 12 dogs housed with confirmed infected individuals in Northern Spain, in April–May 2020 [113]. These data suggest that dogs do not play a major role in COVID-19.

##### Cats

Antibodies were detected in 15/102 (14.7%) of domestic cats sampled in Wuhan, China, after the local SARS-CoV-2 outbreak between January and March 2020, using ELISA and/or neutralization assay. Three cats with the highest titer were owned by three patients, indicating potential direct human-to-cat transmission rather than cat-to-cat transmission. Conversely, sera collected from stray cats or hospital cats had significantly lower titers, and no viral RNA has been detected in nasopharyngeal and anal swabs [114]. In Hong Kong, viral RNA was detected in the oral cavity, nasal and rectal swab samples obtained on March 30, 2020, from a clinically healthy pet cat whose owner was infected with the virus [115], and 14 additional cats from households in which one or more people were ill tested negative. In Belgium, on March 18, viral RNA of SARS-CoV-2 was detected in the feces and vomit of a cat with digestive and respiratory clinical signs. The owner of the cat was also infected with SARS-CoV-2, suggesting human-to-cat transmission [116]. In New York City, USA, on April 22, two pet cats were confirmed positive in two separate locations. Both cats had mild respiratory signs. Human-to-cat transmission has been suggested as a source of infection for both cats [117]. In Northern Spain, one out of eight cats tested positive for SARS-CoV-2 RNA in nasal swabs in April–May 2020. The cat was housed with an infected patient with severe COVID-19 symptoms [113]. Other limited surveillances in cats revealed neither RNA nor antibodies in pet cats in residence with infected individuals in France [112]. These findings revealed that pet cats are more susceptible than dogs for SARS-CoV-2. They may develop mild symptoms and excrete the virus. Whether cats can play a role in virus transmission to humans or other animals is not yet clear. 

##### Tigers

A four-year-old female Malayan tiger in the Bronx Zoo in New York City, USA, tested positive in April 2020. The virus was detected in respiratory-tract samples. The tiger exhibited respiratory signs (i.e., dry cough). Four more tigers in the zoo tested positive. Other co-housed tigers and animals tested negative, assuming poor animal-to-animal transmission. The infection was presumably acquired by an asymptomatically infected zookeeper [118].

##### Lions

Three African lions in the Bronx Zoo in New York City, USA, tested positive in April, 2020. The animals had a dry cough and inappetence. The infection was probably acquired from an infected yet asymptomatic zookeeper [118]. 

##### Minks

In The Netherlands, minks in two separate farms in Beek en Donk (n = 7500 minks) and in Milheeze (n = 13,000 minks) developed respiratory and gastrointestinal disorders in April 2020. The mortality rate was 1.2 to 2.4%, and deaths were mainly observed in pregnant females. Most of necropsied minks had lung lesions, including interstitial pneumonia. With the use of RT-qPCR, viral RNA was detected in different samples, including the conchae, lung, throat swab, rectal swab and, less frequently, from the liver and intestines. No viral RNA was detectable in the spleens. Some of the workers at the farm had previously tested positive for the SARS-CoV-2; therefore, human-to-animal transmission was the most likely scenario for the infection of minks. Nevertheless, the preliminary sequencing data suggested mink-to-human transmission for one worker; however, investigations are still ongoing [119].

##### Other Animals

Viral RNA was not detected in samples obtained from a guinea pig or two rabbits housed with humans with confirmed COVID-19 infections in three households in Northern Spain, in April–May 2020 [113]. There are knowledge gaps on the role of other animals, particularly cattle, sheep, goats, horses and donkeys, in COVID-19, which should be determined by targeted surveillance. 

#### 2.1.3. Experimental Animal Hosts

Model animals are of imminent importance to understand the pathobiology and amelioration of diseases. Faithful animal models should mimic human disease in sharing comparable morbidity, mortality and route of infection [120]. It is not always possible to find a faithful animal model to recapitulate the pathogenesis of virus infection in humans and to evaluate potential medical countermeasures, including antivirals and vaccines. Although the nonhuman primates (NHP) are the gold-standard for studying emerging viruses in humans [121], they are expensive and difficult to handle, and for ethical reasons (e.g., animal welfare [122]), they are not used as a first-line model. Small animals are easy to handle, cheaper than NHP and commercially available [121]. However, they vary in their susceptibility to different viruses and do not always recapitulate the clinical disease in humans, due to biological variations (e.g., presence of receptors and immune system). For instance, for the emerging CoVs in humans, mice, ferrets and hamsters were susceptible to SARS-CoV infection [123,124,125,126], but not for MERS-CoV [127,128,129], mostly due to species-variations in DPP4 receptors [127,129]. In the last few months, several animal models have been studied to assess the virulence and pathogenesis of different SARS-CoV-2 isolates from different countries. These experiments are summarized in Table 3. 

##### Rhesus Macaques

Rhesus macaques inoculated with a combination of intratracheal (IT), intranasal (IN), ocular (OC) and oral (OR) routes were described by Munster et al. [130]. Macaques showed a transient elevation in body temperature for one day only. In addition to bodyweight loss, some macaques showed changes in the respiratory pattern and piloerection, reduced appetite, hunched posture, pale appearance and dehydration. They completely recovered between 9 and 17 days post-inoculation (dpi). Pulmonary infiltrates were seen by radiographs from 1–12 dpi, which completely resolved by day 14 PI. Postmortem examination revealed interstitial pneumonia. Viral loads have been detected in nose, throat, and anal samples for up to 17 dpi. Viral RNA has been detected in the lungs and respiratory tract, GIT and lymphoid tissues. No viral RNA in the blood or urine samples has been detected. Viral antigen has been detected in the macrophage in the lungs and in the lymph nodes. All animals seroconverted at 10 dpi [130]. 

IT inoculation of six male and female rhesus macaques with SARS-CoV-2 was described by Shan et al. [131]. Only one of the macaques exhibited transient inappetence, and the other animals remained healthy. Viral RNA has not been detected in the blood. Viral RNA has been detected in high amounts at 1 and 5 dpi in oropharyngeal swabs. Likewise, anal swabs have been tested positive in three of the six monkeys. Chest X-ray examination revealed patchy and progressed to multiple glass-ground opacity. Lungs of euthanized monkeys had a variable degree of consolidation, edema, hemorrhage and congestion with interstitial pneumonia. The virus has been re-isolated from the trachea, bronchus and lungs, in addition to the oropharyngeal swabs [131]. 

In another study, IT inoculation of three-to-five-year-old rhesus macaques with SARS-CoV-2 resulted in reduced bodyweight in three out of four monkeys and transient inappetence, tachypnea and hunched posture [132]. Viral loads have been detected in the nasal, oral and anal swabs. Viral RNA has been in the nose, lung, gut, spinal cord, heart, skeletal muscles and bladder. X-ray radiography showed bilateral ground-glass opacification of the lungs, and necropsy at 7 dpi revealed mild to moderate interstitial pneumonia. SARS-CoV-2 antibodies have been detected in sera collected at 14, 21 and 28 dpi. Interestingly, after 28 days post-infection, the monkeys were re-challenged with SARS-CoV-2. Neither viral RNA in different organs nor elevation in antibody titers have been observed, and chest X-rays were normal, indicating full protection from reinfection [132]. 

Moreover, rhesus macaques have been used for the evaluation of inactivated vaccines against SARS-CoV-2 [133]. Intra-tracheal-inoculated non-vaccinated macaques developed severe interstitial pneumonia, and SARS-CoV-2 RNA has been detected in the oral and anal swabs, as well as in the lungs, at 3–7 dpi [133]. These data confirm that rhesus macaques are a faithful animal model for studying the pathogenesis of and vaccine efficacy against SARS-CoV-2 resembling SARS-CoV and MERS-CoV.

##### Ferrets 

The experimental infection of ferrets has been described in several studies. Shi et al. [134] assessed virulence and transmission of two viruses: one from the environmental sample collected in the Wuhan Seafood Market, and another from a patient in Wuhan. IN-inoculated ferrets excreted infectious viruses in the upper respiratory tract (i.e., nasal turbinate, soft palate and tonsils), the virus has not been detected in the trachea, lungs, heart, liver, spleen, kidneys, pancreas, small intestine and brain. In separate experiments, the viral RNA has been detected in the rectal swabs, although at lower levels than those in the nasal washes. No infectious virus has been isolated from rectal swabs of any ferret. Only two ferrets had fever and loss of appetite at day 10 and 12 after infection. All ferrets possessed serum anti-SARS-CoV-2 antibody, using ELISA and serum neutralization test (SNT). In a third experiment by the same team, viral RNA was detected in the nasal turbinate, soft palate, tonsil, and/or trachea for up to 8 dpi in IT-inoculated ferrets. 

Another study has been done by Kim et al. [135]. In this study, IN-inoculated ferrets with a Korean virus exhibited reduced activity, elevated body temperature and occasionally cough. Viral RNA has been detected in the serum, nasal washes, saliva, urine, feces, nasal turbinate, trachea, lungs, intestine and kidneys. Viral antigen has been detected in the nasal turbinate, trachea, lungs and intestine, and acute bronchiolitis has been observed at necropsy. The virus was successfully transmitted to co-housed ferrets (direct contact) and via airborne (indirect contacts), as indicated by the presence of antibodies, using SNT and viral excretion in the nasal washes, saliva, urine and fecal samples for up to 7 days post-exposure [135].

The study conducted by the Erasmus Medical Centre, using ferrets as a model, has been published in a preprint [136]. Ferrets were inoculated intranasally with a German SARS-CoV-2 and after six hours of inoculation-naïve ferrets were co-housed with each inoculated ferret, to assess direct virus contact transmission. At 24 hpi, additional ferrets were housed in a separate cage, to assess airborne transmission. Viral RNA has been detected in inoculated ferrets for up to 19 dpi in the throat, nasal and/or rectal swabs. Likewise, all direct-contact ferrets excreted viruses for up to 17 days post-exposure, and the virus was successfully transmitted by air to three of the four of the indirect-contact ferrets. In the latter group, SARS-CoV-2 RNA was first detected from three to seven days post-exposure, and ferrets remained positive for 13 to 19 days post-exposure [136]. Generally, excretion of the virus from the nasal swabs was higher than in the throat and rectal swabs. Viable viruses have been isolated from the nasal and throat swabs, but not from the rectal swabs. All ferrets seroconverted at 21 dpi with similar levels of antibody in primarily inoculated, direct-contact and most of the indirect-contact ferrets [136]. 

Another study conducted at Friedrich-Loeffler-Institut (FLI), Germany, showed that a German SARS-CoV-2 can efficiently replicate and transmit to co-housed ferrets, without showing clinical signs [137,138,139]. Viral RNA has been detected in the nasal washes, and to a lesser extent in the rectal swabs obtained from inoculated and in-contact ferrets. Moreover, viral RNA has been detected in the respiratory tract, intestine, muscle, skin, lymph node, adrenal gland and/or brain tissues in euthanized inoculated ferrets. Lesions were mostly restricted to the nasal cavity. All inoculated and some co-housed ferrets developed antibodies against SARS-CoV-2 [139].

Together, these experiments have shown that ferrets are a suitable animal model for studying the pathogenesis of SARS-CoV2. They mimic the mild clinical signs of SARS-CoV-2, lung lesions and transmission in humans.

##### Mice

Several studies have been conducted in wild and transgenic mice. Studies showed that SARS-CoV–2 exhibited binding for human ACE2 receptors (hACE2), but limited binding to murine ACE2 [140,141,142,143]. Transgenic mice expressing hACE2 receptors for SARS-CoV–2 viruses were used in one study [144]. IN inoculation of specific-pathogen-free, 6–11-month-old, WT-HB-01 mice and hACE2 mice with SARS-CoV-2 has been done. Only hACE2 transgenic mice exhibited slight bristles and up to 8% weight loss at 5 dpi. Virus isolation and/or detection was successful in the lungs in samples taken from 1 to 7 dpi. Lung lesions and histopathological changes, including pneumonia and infiltration of inflammatory and immune cells, have been described. No remarkable histopathological changes or viral antigens in the myocardium, liver, spleen, kidney, cerebrum, intestine and testis have been observed [144]. In another study, 17-week-old transgenic female C57Bl/6 Ces1c^-/-^ mice were inoculated intranasally with chimeric SARS-CoV carrying SARS2-RdRp. Mice developed bodyweight loss and lung hemorrhages and dysfunction. The virus has been isolated from the lungs at 5 dpi [145].

Another study compared the infectivity of a Belgian SARS-CoVs-2 in wild-type BALB/C mice and transgenic mice lacking functional T and B cells. The virus replicated at similar levels in both mice breeds, without remarkable differences in lung pathology. These results indicated that SARS-CoV-2 replicated, although at low levels, in mice lacking hACE2 [146]. Moreover, wild-type (WT) C57BL/6 mice and C57BL/6 mice with genetic ablation of their type I (Ifnar1^-/-^) and III interferon (IFN) receptors (Il28r^-/-^) were inoculated IN with SARS-CoV-2. Increased replication of the virus in the lungs was observed in Ifnar1^-/-^ mice 3 dpi, compared to WT and Il28r^-/-^ mice. Moreover, Ifnar1^-/-^ mice exhibited increased levels of intra-alveolar hemorrhage, sometimes with peribronchiolar inflammation. Interestingly, pretreatment of Ifnar1^-/-^ mice with human convalescent SARS-CoV-2 patient serum reduced viral loads in the lungs [146]. These findings indicate that the transgenic mice, not wild-type mice, may play an important role in studying the immunopathology of COVID-19.

##### Hamsters

Many studies described the infection of SARS-CoV-2 in hamsters. In the first study, golden Syrian hamsters, 6–10 weeks old, were IN-inoculated with SARS-CoV-2 isolated from the nasopharyngeal aspirate of a patient in Hong Kong, after propagation in VeroE6 cells [147]. Primarily inoculated animals developed clinical signs within one week post-inoculation, including lethargy, ruffled fur, hunched back posture, tachypnea and ~11% loss of bodyweight. None of the animals died. Viral RNA was detected in the nasal turbinate and trachea from 2 to 7 dpi. Virus load was high in the lungs, and lower levels were detected in the intestine, salivary glands, heart, liver, spleen, lymph nodes, kidney, brain and blood particularly at 4 dpi. Hamsters recovered at 14 dpi and showed high serum neutralizing antibodies at 7 and 14 dpi. Euthanized hamsters showed pathological changes in the nasal turbinate, trachea and lungs, including lung consolidation and severe pulmonary hemorrhage. Viral N-protein was observed in the lungs and intestine. In the lungs, induction of interferon-γ and pro-inflammatory chemokines/cytokines were described. Viral transmission to naïve co-housed hamsters was successful. Although in-contact hamsters did not suffer reduction in bodyweight gains, the histopathological changes and viral expression in nasal turbinate, trachea, lung and extra-pulmonary tissues were similar to those of the primarily inoculated hamsters. Moreover, passive immunization of hamsters significantly reduced viral loads in the nasal turbinate and lungs; however, this occurred without significant impact on clinical signs or histopathological changes [147]. 

In a second study, four–five-week-old male golden Syrian hamsters were intranasally inoculated with SARS-CoV-2 virus [148]. Hamsters had ruffled hair coat. Viral RNA was detected from 2 to 14 dpi, with the highest viral load in the lungs and to lower levels in the kidneys and from fresh fecal samples. At necropsy, pneumonia and lung consolidation were reported. Viral N-protein was demonstrated in the nasal epithelial cells, lungs and duodenum. Viral clearance and tissue repair were observed on 7 dpi. The virus transmitted efficiently from the primarily inoculated hamsters to co-housed naïve hamsters. The inoculated hamsters and co-housed hamsters lost > 10% of the bodyweight. Viral RNA was detected in the nasal washes obtained 3 dpi from co-housed hamsters. All hamsters recovered, and neutralizing antibodies were detected within 14 dpi. 

In a third study, seven-to-eight-week-old golden Syrian hamsters (males and females) were challenged IN with SARS-CoV-2 WT or a mutant SARS-CoV-2 virus with a deletion of the polybasic CS [149]. The WT virus caused more extensive histopathological changes in the lungs of infected animals and replicated more efficiently in the tracheal and lung tissues than the variant virus [149]. 

Another study compared the susceptibility of WT and STAT2-/- or IL28R-a -/- transgenic hamsters with ablated Signal Transducer and Activator of Transcription 2 (STAT2-/- lacking type I and III IFN signaling) and IL28R expression (IL28R-a - /- lacking IFN type III signaling) [146]. After IN-inoculation with a Belgian virus, all wild-type hamsters had high viral loads in the lungs, with multifocal necrotizing bronchiolitis, massive leukocyte infiltration and edema. STAT2-/- hamsters developed high viral load in the lungs, high titer viremia, high levels of viral RNA in the spleen, liver and upper and lower gastrointestinal tract (GIT) and less-severe lung pathology. These data indicate that STAT2 plays a role in SARS-CoV-2 pathogenesis, by restricting the systemic spread of the virus, yet it increases lung pathology [146].

Taken together, these experiments showed that hamsters are a valuable small animal model to study the pathogenesis, immunopathology and transmission of SARS-CoV-2.

##### Dogs

Three-month-old beagles have been challenged IN, using a Chinese virus, to assess virus replication and transmission [134]. Viral RNA has been detectable in the rectal swabs; however, no viral RNA was detectable in any organ or tissue collected from a euthanized dog at 4 dpi. No infectious virus has been recovered, and two of the inoculated dogs seroconverted, using ELISA. Neither antibodies nor virus has been detected in cohoused dogs, indicating low susceptibility of dogs to SARS-CoV-2 [134].

##### Cats 

Replication and transmission of a Chinese SARS-CoV-2 in subadult cats (aged six-to-nine months) after IN challenge have been studied [134]. At 3 dpi, viral load was evident in the nasal turbinate, soft palates, tonsils, tracheas, lungs and small intestine of euthanized cats. Moreover, the virus was transmitted aerogenically to other cats, and the viral RNA has been detected in the fecal samples. Seroconversion and neutralizing antibodies have been detected in inoculated and exposed cats and severe lesions in the upper and lower respiratory tracts, including the lungs, have been recorded [134]. Likewise, IT, IN, OC and OR inoculation of 15–18-week-old male and female domestic cats with SARS-CoV-2 and virus transmission to naïve cohoused cats has been recently described [150]. Cats did not exhibit clinical signs, although viruses have been isolated in the nasal swab specimens 1 to 6 dpi from inoculated cats and 3 and 9 dpi from cohoused cats. Virus detection was not successful in the rectal swabs. All cats seroconverted at 24 dpi [150]. Those two experiments further confirm that cats are more susceptible than dogs to SARS-CoV-2. It remains to be studied the potential role of cats in the transmission of the virus to other mammals.

##### Pigs

To date, two studies determined the susceptibility of pigs to the infection and transmission of different SARS-CoV-2 isolates [134]. After IN-challenge, neither viral RNA nor antibodies have been detected in inoculated animals [134,139] or in naïve contact pigs [134]. These experiments suggest that pigs are not vulnerable to SARS-CoV-2.

##### Tree Shrew

Experimental infection of male and female tree shrews of different ages, ranging from six months to seven years, with SARS-CoV-2, has been described [151]. After IN-inoculation, most animals, particularly females, showed an increase in body temperature, without showing clinical signs or gross lesions. Viral RNA has been detected, particularly in the younger animals, for up to 12 dpi, in the nasal, throat and anal swabs and/or the blood samples. The RNA has been detected in different organs, including the lungs, pancreas and uterus. Pathological alterations have been observed mainly in the lungs, and to a lesser extent in other organs, including the spleen, intestine, brain, liver and heart [151]. 

##### Bats

The susceptibility of Egyptian fruit bats, which are genetically and immunologically distinct from the putative reservoir horseshoe bats [152,153], was studied after IN-inoculation with a German SARS-CoV-2 [137,139]. Despite not showing any clinical symptoms, the bats excreted viruses orally for up to 12 dpi. Moreover, viral RNA and/or infectious virus was detected in respiratory tissues and at lower levels in other organs, including the heart, skin and intestine [139]. Anti-SARS antibodies were detected in inoculated and contact bats. Viral RNA was detected in co-housed bats for up to 21 dpi, indicating successful bat-to-bat transmission [139]. The results of this experiment further indicate that bats play a role in the replication and transmission of SARS-CoV-2. 

##### Poultry

The susceptibility of poultry to SARS-CoV-2, using different genetically distinct viruses, has been described. Replication and transmission of SARS-CoV-2, Wuhan strain, in chickens showed that neither RNA nor antibodies were detectable at 14 dpi [154]. Likewise, chickens inoculated with a German strain did not develop clinical signs, lesions or antibodies [139]. Furthermore, neither IN-inoculated nor cohoused ducks excreted viral RNA in swab samples, and all of the animals were seronegative 14 dpi [154]. Likewise, chickens, ducks, turkeys, quail and geese challenged with SARS-CoV-2 did not show any clinical signs, and no virus replication or antibodies have been detected [155]. These experiments suggest that poultry are not susceptible to the virus, and it is unlikely that they play a role in COVID-19.

## 3. Summary and Conclusion 

**Figure 1:**
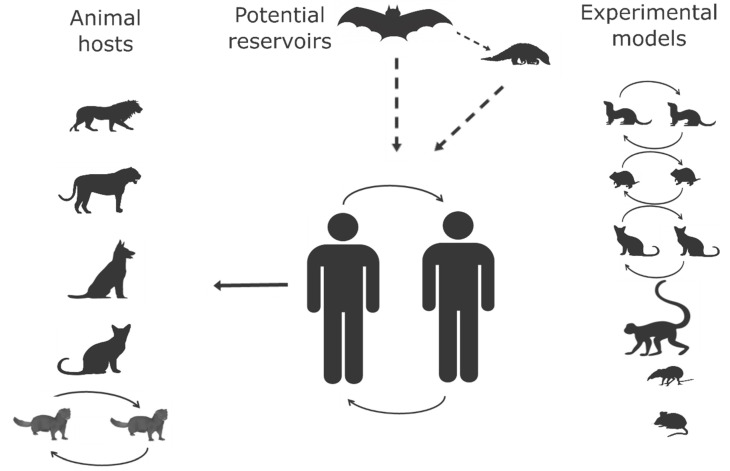
SARS-COV-2 and animal hosts.

COVID-19 is the first known pandemic caused by a coronavirus, and SARS-CoV-2 is the third virus in this family to cause fatal infections in humans, after SARS-CoV and MERS-CoV. Animals are involved in COVID-19 as reservoirs, animal hosts and experimental models (Figure 1). The virus originated from an animal reservoir, most likely bats and/or pangolins or a yet-to-be-identified animal host. Targeted and retrospective surveillance should be extensively done to identify the reservoirs for SAR-CoV-2 and other related viruses before they transmit to humans. 

There are no data available on systemic surveillance, particularly in farm animals; however, it is likely that SARS-CoV-2 will be established in human populations and not in animals. There are several reasons for this assumption. (1) CoVs evolve at a lower rate than other RNA viruses (e.g., influenza), due to the proofreading of RdRp. Therefore, it is less likely to be established in other animals. (2) SARS-CoV-2 shares similarities with SARS-CoV, which had a limited natural host-range, including cats and raccoon dogs, and has been occasionally reported in other animals [161,162]. (3) So far, there is no evidence that HCoV-OC43 has been reported in animals, although it was transmitted from cattle-to-humans around 1890 [20]. (4) Fortunately, many domestic and companion animals are less susceptible to SARS-CoV-2 compared to humans. The low susceptibility of animals is probably attributed to restricting host-factors, e.g., functional ACE2 and specific proteases. A recent study has shown that the proportions of cells carrying both ACE2 and TMPRSS2 were high in cats, low in pigs, very rare in dogs and absent in chickens [163]. (5) To date, anthroponotic transmission is the main pathways for the infection and fatalities caused by SARS-CoV-2 in few companion and zoo animals, and no strong evidence for natural animal-to-human transmission, except for mink, which remains to be confirmed. Importantly, there is no sustained animal-to-animal transmission. (6) Last but not least, many CoVs are endemic in animals in several countries, and no clear evidence is available for the transmission to humans. Moreover, whether the immune response against CoVs in animals can confer some protection against SARS-CoV-2 remains to be studies.

To understand the pathobiology of the virus, experimental infections have been conducted in several animal species. Results showed that rhesus macaques, hamsters, ferrets, cats and fruit bats were permissive, while dogs, pigs and poultry were resistant. Monkeys (e.g., rhesus macaques) developed mild-to-moderate clinical signs, as seen in the majority of human SARS-CoV-2 infections; however, they are expensive and difficult to handle and are not available in each lab. Hamsters and ferrets seem to be the most suitable models to study the molecular pathobiology of SARS-CoV-2 similar to SARS-CoV [124], but not to MERS-CoV [128], probably due to different receptors (ACE2 for SARS-viruses vs. DPP4 for MERS-CoV) [148]. So far, ferrets, hamsters, cats and, to a lesser extent, bats, were used to assess animal-to-animal transmission. Moreover, wild-type mice are a poor model to assess virus pathogenesis or antiviral and vaccine efficacies. However, transgenic mice are a model that can be considered, particularly to study the elements of the immune system, which might confer resistance to SARS-CoV-2 infections. 

CoVs infection in humans was neglected for years. The recurrent severe infections of animal-coronaviruses in the last two decades indicate that future outbreaks of related or unrelated CoVs in humans are inevitable. Although difficult to be achieved, there is an urgent need to develop universal vaccines and antivirals against CoVs. Currently, there are several potential vaccines and antivirals against SARS-CoV-2, and some of them are under evaluation in clinical trials [164,165]. Although the limited resources may prevent the wide application of vaccines against SARS-CoV-2 in animals, evaluation of vaccines or antivirals should be considered for susceptible animals (i.e., pets and zoo animals). Vaccination of reservoir animals against rabies virus (an RNA virus) has proven to be effective to control rabies virus infections in humans and animals, and have allowed the eradication of rabies in terrestrial carnivores in several regions worldwide [166].

## Figures and Tables

**Table 1:** Major members of family Coronaviridae and their receptors.

Genus	Subgenus	Species	Receptor	Reference
*Alphacoronavirus*	*Pedacovirus*	Porcine epidemic diarrhea virus	APN	[24]
	*Duvinacovirus*	Human coronavirus 229E	APN	[25]
	*Setracovirus*	Human coronavirus NL63	ACE2	[26]
	*Rhinacovirus*	Swine acute diarrhea syndrome coronavirus	NI	
	*Tegacovirus*	Alphacoronavirus 1		
		Canine coronavirus	APN	[27]
		Feline infectious peritonitis virus	APN	[28]
		Porcine transmissible gastroenteritis virus	APN	[29]
		Porcine respiratory coronavirus	APN	[30]
*Betacoronavirus*	*Embecovirus*	Betacoronavirus 1		
		Human coronavirus OC43	Neu5,9Ac2	[31]
		Equine coronavirus	NI	
		Bovine coronavirus	Neu5,9Ac2/ HLA-I	[32,33]
		Dromedary camel coronavirus HKU23	Sugar	[34]
		Canine respiratory coronavirus	HLA-I	[33]
		Porcine hemagglutinating encephalomyelitis virus	Neu5,9Ac2	[35]
		HCoV-HKU1	Neu5,9Ac2	[36]
		MHV	CEACAM1^a^	[37]
	*Merbecovirus*	MERS-CoV	DPP4	[38]
	*Sarbecovirus*	SARS-CoV-1	ACE2	[39]
		SARS-CoV-2	ACE2	[21]
*Gammacoronavirus*	*Igacovirus*	Avian infectious bronchitis virus	Neu5Gc	[40]
		Turkey coronavirus (TCoV)	non-sialylated type 2 poly-LacNAc	[41]
*Delatacoronavirus*	*Buldecovirus*	porcine deltacoronavirus	NI	

NI = not identified.

**Table 2:** Genome organization and S1/S2 cleavage site of different human coronaviruses.

Species	Genome Organization	S1/S2
HCoV-229E	5’UTR-Rep-**S**-3a-3b-**E**-**M**-**N**-3’UTR	DGSIIAVQPR↓NVSYD
HCoV-NL63	5’UTR-Rep-**S**-3-**E**-**M**-**N**-3’UTR	DGSLIPVRPR↓NSSDN
HCoV-OC43	5’UTR-Rep 2-**HE**-S-5-**E**-**M**-**N**-3’UTR	VDYSKNRRSR↓GAITT
HCoV-HKU1	5’UTR-Rep-**HE**-**S**-4-**E**-**M**-8-**N**-3’UTR	SSSSSRRKRR↓SISA
MERS-CoV	5’UTR-Rep-**S**-3-4a-4b-5-**E**-**M**-8b-**N**-3’UTR	PSTLTPRSCR↓SVPG
SARS-CoV-1	5’UTR-Rep-**S**-3a-3b-**E**-**M**-7a-7b-8a-8b-9b-**N**-3’UTR	TVSL....LR↓STGQ
SARS-CoV-2	5’UTR-Rep-**S**-3a-**E**-**M**-6-7a-7b-8-**N**-3’UTR	TQTNSPRRAR↓SVAS

Black bold letters are structural ORFs, while red bold letters refer to the furin cleavage site. HCoV-229E, HCoV-NL63 and SARS-CoV lack furin-like cleavage site.

**Table 3:** Experimental animal models for SARS-CoV-2.

Animal	Age, Route, Dose	Virus	Symptoms	Pathology, Immunology (Major Changes)	Replication	Seroconversion	References
Monkey	*Macaca mulatta*, adults–old, 4.75 × 10^6^ pfu, IT, IN, OC.	Chinese strain	Elevated body temperature (> 38 °C), decreased bodyweight	Lung radiographic abnormalities, severe gross lesions on lung, heart and stomach and inflammation in liver and heart. Transient increase in blood CD4+ T cells, CD8+ T cells, monocytes. Increased cytokine response	RNA in nasal, throat and anal swabs, blood, fecal samples	>4 dpi	[156]
	Young *M. mulatta*, ~2.3 × 10^6^ pfu, IT, IN, OC.		Elevated body temperature (> 38 °C), decreased bodyweight	Lung radiographic abnormalities, severe gross lesions on lung, heart and stomach, and inflammation in liver and heart Transient increase in blood CD4+ T cells, CD8+ T cells, monocytes. Increased cytokine response	RNA in nasal, throat and anal swabs, blood, fecal samples	>4 dpi	[156]
	*Macaca fascicularis*, 4.75 × 10^6^ pfu, IT, IN, OC.		Transient elevated body temperature (> 38 °C), decreased bodyweight	Lung radiographic abnormalities, severe gross lesions on lung, heart and stomach and inflammation in liver and heart Transient increase in blood CD4+ T cells, CD8+ T cells, monocytes. Increased cytokine response	RNA in nasal, throat and anal swabs, blood, fecal samples	>4 dpi	[156]
	*Callithrix jacchus,* 10^6^ pfu, IN.		No signs	No severe lesions	RNA in nasal, throat and anal swabs, blood	Negative	[156]
Cynomolgus Macaques	4–5 yr, IT and IN, 10^6^ TCID_50_	BetaCoV/Munich/BavPat1/2020	No signs	Consolidated pulmonary tissues	RNA in nasal swabs, nasal cavity, trachea, bronchi, lungs, ileum, tracheo-bronchial lymph nodes, tonsils	14 dpi	[157]
	15–20 yr, IT and IN, 10^6^ TCID_50_		One animal had serous nasal discharge	Consolidated pulmonary tissues	RNA in nasal swabs, nasal cavity, trachea, bronchi, lungs, ileum, tracheo-bronchial lymph nodes, tonsils	14dpi	[157]
Rhesus Macaques	2.6 × 10^6^ TCID_50,_ IT, IN, OC and OR	nCoV-WA1-2020	Changes in respiratory pattern, piloerection, reduced appetite, hunched posture, pale appearance and dehydration	Lung infiltrates by radiographs, interstitial pneumonia, pulmonary edema Leukocytosis, neutrophilia, monocytosis lymphopenia, increased cytokine and chemokines	Nose, throat, rectal swabs, lungs, bronchioalveolar lavage, lymph nodes, GIT tissues	>10 dpi	[130]
	2.6 × 10^6^ TCID_50,_ IN, OR, OC and IT	nCoV-WA1-2020	Increased respiratory rate, difficulty breathing	Lung infiltrates by radiographs			[158]
	3–5yr, 10^6^ TCID_50_, IT	WH-09/human/2020/CHN	Bodyweight loss, transient inappetence, tachypnea and hunched posture, bilateral ground-glass opacification of the lungs	Mild-to-moderate interstitial pneumonia	Viral RNA was detected in nasal, oral and anal swabs, as well as in the nose, lung, gut, spinal cord, heart, skeletal muscles and bladder	>14 dpi	[132]
	10^6^ TCID_50_ IT	CN1, Chinese virus		Severe interstitial pneumonia	RNA in pharynx, crissum, lung, anal swabs by day 3–7 dpi		[133]
	6–12 yr, 7 × 10^6^ TCID_50_ IT	IVCAS 6.7512- Wuhan strain	Reduced appetite, bodyweight loss	Interstitial pneumonia	Virus was isolated from oropharyngeal swabs, trachea, bronchus, lungs	14 and 21 dpi	[131]
	3–5 yr, 10^6^ TCID_50_, IN	BetaCoV/Wuhan/IVDC-HB-01/2020	Weight loss, asthenia	Radiographic changes (ground-glass opacities), interstitial pneumonia Declined CD3+/CD8+ and CD3+/CD4+ T cells	Viral RNA in nasal, throat and anal swabs and lungs	14 dpi	[159]
	15 yr, IN, 10^6^ TCID_50_		Weight loss, asthenia	Radiographic changes (ground-glass opacities), severe interstitial pneumonia	Viral RNA in nasal, throat and anal swabs and lungs	14 dpi	[159]
	3–5 yr, 10^6^ TCID_50_ IT	WH-09/human/2020/CHN	Weight loss	Radiographic changes, moderate interstitial pneumonia	Viral RNA in anal swab		[160]
	3–5 yr, 10^6^ TCID_50_ OC		No signs	Mild interstitial pneumonia, mild lung lesions	High viral RNA in conjunctival swab		[160]
	3–5 yr, 10^6^ TCID_50_, IG		No signs	Radiographic lung changes,	No viral load		[160]
Ferrets	10^5^ PFU, IN or IT	F13/environment/2020/Wuhan, (F13-E), and SARS-CoV-2/CTan/human/2020/Wuhan (CTan-H)	Fever and inappetence	Severe lymphoplasmacytic perivasculitis and vasculitis, increased numbers of type II pneumocytes, macrophages, and neutrophils, mild peribronchitis	Viral RNA in nasal, throat and anal swabs, nasal turbinate and soft palate		[134]
	**12–20 Mo, IN, 10^5.5^ TCID_50_**	NMC-nCoV02, Isolate from Korean patient 2020	Reduced activity, elevated body temperature and occasionally cough	Acute bronchiolitis, viral antigens in nasal turbinate, trachea, lungs, and intestine Increased immune cell infiltration in the respiratory tract	RNA in serum nasal washes, saliva, urine, feces, nasal turbinate, trachea, lungs, intestine, kidneys	Yes	[135]
	**6 Mo, IN, 6. 10^5^ TCID_50_/0.5mL**	BetaCoV/Munich/BavPat1/2020			Viral RNA in nasal, throat and rectal swabs for up to 19 dpi	21 dpi	[136]
	9–12 Mo, IN,10^5^ TCID_50_	hCoV-522 19/Germany/BavPat1/2020	No signs	Lesions were mostly restricted to the nasal cavity Perivascular lymphocytic infiltration and minimally increased numbers of alveolar macrophages	Viral RNA in nasal washes, rectal swabs, respiratory tract, intestine, muscle, skin, lymph node, adrenal gland and/or brain tissues	> 8 dpi	[139]
Hamsters	6–10 Wk, IN, 10^5^ pfu/0.1 mL	Hong Kong strain	Tachypnea, weight loss	Diffuse alveolar damage with extensive apoptosis, severe lung hemorrhage, Proliferative phase of tissue repair, airway and intestinal involvement with virus N-protein expression, high lung viral load Spleen and lymphoid atrophy associated with marked cytokine activation	Nasal turbinate, trachea, lung, intestine, salivary glands, heart, liver, spleen, lymph nodes, kidney, brain and blood	7 and 14 dpi	[147]
	4–5 Wk, IN, 8 × 10^4^ TCID_50_/80µL	First confirmed COVID–19 Patient in Hong Kong	Ruffled hair coat, bodyweight loss	Pneumonia, lung consolidation CD3 positive T lymphocytes in the peri-bronchial region	Nasal turbinate, lung, kidney, duodenum	14 dpi	[148]
	7–8 Wk, IN, 1.5 × 10^5^ pfu	Hong Kong strain	Bodyweight loss	Extensive alveolar wall destruction, alveolar space hemorrhage and mononuclear cell infiltration in the lungs of	Virus was isolated in the tracheal and lung tissues		[149]
	6–8 Wk, 2 × 10^5^ TCID_50_, IN	Belgium/GHB-03021/2020		Multifocal necrotizing bronchiolitis massive leukocyte infiltration and edema	High viral RNA loads and infectious titers in the lungs		[146]
	Transgenic hamster, 5–12 Wk, 2 × 10^5^ or 2 × 10^6^ TCID_50_, IN	Belgium/GHB-03021/2020		Limited infiltration of polymorphonuclear leukocytes	High viral RNA loads and infectious titers in the lungs, blood, spleen, liver and upper and/or lower GIT		[146]
Mice	BALB/C, 2 × 10^5^ TCID_50_, IN	Belgium/GHB-03021/2020		Mild lung pathology upregulation of antiviral effector molecules	Low-level replication in the lungs		[146]
	SCID mice (lacking functional T and B cells), 2 × 10^5^ TCID_50_, IN	Belgium/GHB-03021/2020		Mild lung pathology	Low-level replication in the lungs		[146]
	WT, or Il28r-/- C57BL/6 mice, 2 × 10^5^ TCID_50_, IN	Belgium/GHB-03021/2020		Mild lung pathology	Low-level replication in the lungs		[146]
	Ifnar1-/- C57BL/6 mice, 2 × 10^5^ TCID_50_, IN	Belgium/GHB-03021/2020		Increased levels of intra-alveolar hemorrhage, peribronchiolar inflammation	enhanced replication in the lung on 3 dpi		[146]
	6–11 Mo, IN, 10^5^ TCID_50_/50 μL	BetaCoV/Wuhan/IVDC-HB169 01/2020	WT-HB-01, no signs	No histopathological changes	No virus was detected in the lungs		[144]
	Transgenic mice, 6–11 Mo, IN, 10^5^ TCID_50_/50 μL	BetaCoV/Wuhan/IVDC-HB169 01/2020	hACE2 mice, slight bristles, bodyweight loss	Lung discoloration, damaged, swollen, enlarged, pneumonia with accumulation of lymphocytes and monocytes, macrophages, T and B lymphocytes	Viruses were isolated/detected from the lungs	21 dpi	[144]
	Transgenic mice, 17-week, IN, 10^3^ PFU	2019-nCoV/USA-WA1/2020	Female C57Bl/6 Ces1c^-/-,^ bodyweight loss	Lung hemorrhages and dysfunction	Viruses were isolated from the lungs		[145]
Tree Shrew	6–12 Mo, 2–4 yr, 5–7 yr, IN, 10^6^ PFU	Transient elevated body temperature		Pathological alterations in lungs, intestines, spleen, brain, heart, liver, pancreas	Viral RNA in nasal, throat, anal swabs and/or blood, lungs, pancreas, uterus,		[151]

IT = intratracheal, IN = intranasal, OC = ocular, OR = oral, IG = intragastric, Wk = week, Mo = month, yr = year, pfu = plaque forming units, TCID50 = mean tissue culture infective dose, GIT = gastrointestinal tract. Studies highlighted in gray confirmed direct animal-to-animal transmission and those written in bold confirmed also airborne transmission.
